# Bio-products from *Serratia marcescens* isolated from Ghanaian *Anopheles gambiae* reduce *Plasmodium falciparum* burden in vector mosquitoes

**DOI:** 10.3389/fitd.2022.979615

**Published:** 2022-09-29

**Authors:** Esinam Abla Akorli, Prince Chigozirim Ubiaru, Sabyasachi Pradhan, Jewelna Akorli, Lisa Ranford-Cartwright

**Affiliations:** 1 Department of Parasitology, Noguchi Memorial Institute of Medical Research, University of Ghana, Legon Accra, Ghana; 2 School of Biodiversity, One Health and Veterinary Medicine, College of Medical, Veterinary and Life Sciences, University of Glasgow, Graham Kerr Building, Glasgow, United Kingdom

**Keywords:** mosquito midgut microbiota, bacterial bio-products, *Enterobacter cloacae*, *Serratia marcescens*, *Anopheles gambiae s.l*, *Plasmodium falciparum*, disease transmission blocking

## Abstract

Novel ideas for control of mosquito-borne disease include the use of bacterial symbionts to reduce transmission. Bacteria belonging to the family Enterobacteriaceae isolated from mosquito midgut have shown promise in limiting *Plasmodium* intensity in the *Anopheles* vector. However, the mechanism of interaction between bacteria and parasite remains unclear. This study aimed at screening bio-products of two bacteria candidates for their anti-Plasmodial effects on mosquito stages of P. *falciparum. Enterobacter cloacae* and *Serratia marcescens* were isolated from field-caught *Anopheles gambiae* s.l. Spent media from liquid cultures of these bacteria were filtered, lyophilized and dissolved in sterile phosphate buffered saline (PBS). The re-dissolved bacterial products were added to gametocytaemic blood meals and fed to *An. gambiae* mosquitoes *via* membrane feeders. Control groups were fed on infected blood with or without lyophilized LB medium. The effect of the products on the infection prevalence and intensity of *P. falciparum* in mosquitoes was assessed by dissecting mosquito midguts and counting oocysts 10-11 days post-infection. *S. marcescens* bio-products elicited significant reduction in the number of mosquitoes infected (*P*=4.02 x10^-5^) with *P. falciparum* and the oocyst intensity (*P*<2 x 10^-16^) than *E. cloacae* products (*P*>0.05 for both prevalence and intensity) compared to the control (lyophilized LB medium). These data support the use of bioproducts released by *S. marcescens* for malaria control based on transmission blocking in the vector.

## Introduction

Malaria elimination has been on the agenda for decades and various schemes towards its achievement have been implemented especially in sub-Saharan Africa (1). These strategies have focused primarily on reducing *Anopheles* vector contact, or *Anopheles* numbers (2, 3). While the largely chemical-based interventions have led to a significant decline in malaria global burden, the risk of infection and the morbidity and mortality caused by malaria persist (4). Resistance to commonly used insecticides is widespread in the mosquito vector populations which hampers the progressive decline of malaria incidence as reviewed in (5). The paradigm for control and elimination of malaria and other mosquito-borne diseases now includes transmission-blocking mechanisms. Mosquito-endosymbiont interactions have become central to the discovery and development of disease blocking components.

The mosquito midgut is a major bottleneck in the *Plasmodium* parasite life cycle (6). There is a significant decline in the number of parasites during their traversal of the vector midgut, making the site important for exploiting transmission-blocking potential. Ingested parasites share the mosquito midgut environment with symbiotic bacteria (microbiota) (7, 8). Several of these bacteria have shown significant negative impact on *Plasmodium* development (reviewed in (9)) making the mosquito microbiota an integral part of vector competence (8, 10). *Enterobacter* spp. and *Serratia marcescens* isolated from *Anopheles* mosquitoes have shown inhibitory results against *P. falciparum* infection when they are mixed with infected blood and fed to mosquitoes (11, 12).

The anti-parasitic effects of mosquito midgut microbiota is thought to be either through stimulation of the anti-microbial immune responses which are cross-reactive with ingested parasites, or a direct influence on the developing parasites (production of metabolites, enzymes or toxins) (13–15). *An. gambiae* thioester-containing proteins (TEPs) are particularly involved in phagocytosis of some bacteria (16) and implicated in the destruction of *Plasmodium* by binding to the parasite surface and eliciting haemocyte encapsulation (17–19). The interaction between mosquito midgut bacteria and parasites could also be direct, through biomolecules that the microbes produce (12, 20). For example, *Serratia ureilytica* isolated from the midguts of *An. sinensis* block *P. vivax* ookinete to oocyst development *via* lipase activity (15), although this function is not assumed to be ubiquitous among *Serratia* spp (15, 20).

In this study, we investigate the direct anti-parasitic potential of two bacteria species, *E. cloacae* and *S. marcescens*, which are commonly isolated from blood-fed *Anopheles* mosquitoes. Here, the effect on mosquito stages was examined by feeding bacterial bio-products and *P. falciparum* to *Anopheles gambiae s.s.* and assessing the prevalence and intensity of infection.

## Materials and methods

### Preparation of bacterial spent medium

*S. marcescens* (*Sm*) and *E. cloacae* (*Ec*) were previously isolated from the midgut of adult female *An. gambiae* s.l collected from a farm site at Opeibea, Ghana and stored in glycerol at -80°C (21). These were revived by culturing the isolated bacteria species each separately in LB broth overnight at 37°C with shaking at 120rpm and then plating on LB agar to confirm the presence of pure colonies. Bacteria cells were picked and put into culture overnight. Optical density was measured at OD_600nm_ and adjusted to obtain a standardised OD of 0.50-0.55. For each bacterium, 500μL of standardised culture was inoculated into 50mL sterile LB broth. A control tube was set with 50mL sterile LB broth without inoculum. All tubes were incubated as above and retrieved after 8 hours which corresponds to the stationary phase of the two bacteria species (data not shown). Each tube was centrifuged at 18,407 xg to separate the bacteria cells from the spent medium, and supernatant was passed through 0.2μm disc filter to remove cells. The OD of the supernatant pre- and post-filtration was measured and compared to confirm the removal of bacterial cells (Supplementary Table 1). The filtrate was lyophilized by freeze-drying and stored at 4°C until use. Stock concentrations of bioproducts were prepared by dissolving the dry sample in 1X sterile PBS: LB media control (50mg/mL), *S. marcescens* (150mg/mL), *E. cloacae* (100mg/mL). Working concentrations of the dissolved products were freshly prepared on the day of mosquito infection experiments by further diluting with sterile 1X PBS and filtering before mixing with the blood meal to get final concentrations.

### Mosquito strain

*An. gambiae* s.s. Kisumu strain mosquitoes (22) were reared under standard conditions of 26°C, 80% relative humidity and a 12:12 hour light:dark cycle. Adult mosquitoes were maintained on 10% glucose with 0.05% para-aminobenzoic acid (PABA) solution, given *ad libitum*. Fifty (50) 3–5-day old female adults were placed into each of four experimental groups and starved for 24 hours prior to infectious blood feeding at 5-7 days post emergence.

### Infection experiments

*Plasmodium falciparum* clone 3D7 (23) parasites were cultured to produce gametocytes according to standard protocols (24) and used to prepare infectious blood meals. 30μL of re-suspended bacterial product was added to 1.5mL infectious bloodmeal to give a final concentration of 1mg/mL (LB), 2mg/mL (*Ec*) and 3mg/ml (*Sm*). These concentrations were previously identified as effective on asynchronous *Plasmodium* in an *in vitro* assay (data not shown). A second control was prepared with only infectious blood and without any bio-product (control). The gametocyte density in each blood meal was assessed from Giemsa-stained thin blood films made of the blood meal preparation. The infection experiment was performed with three independent replicates. Mosquitoes were dissected 10-11 days after the infectious feed and the midguts were examined at 400x magnification to establish the number of mosquitoes infected (prevalence) and oocyst number (intensity) in each infected mosquito.

### Estimation of mosquito body size

The mosquito body size was measured to allow consideration as a likely determinant of parasite prevalence and intensity. The wing length of the mosquito was measured as an indicator of mosquito body size (25, 26). A wing was removed from each mosquito and the length from the axillary incision to the apical margin was measured using a digital camera imaging system (Moticam 2300) connected to a microscope eyepiece and pre-calibated software (Motic images plus v2.0).

### Statistical analyses

Data obtained from this study were analyzed using generalized linear mixed models in R version 4.0.4. Generalized Linear Mixed Models (GLMM) were used to determine the significant factors explaining the differences in prevalence and infection intensity. The mixed models included treatment with re-suspended product, mosquito body size, blood meal gametocyte density, and replicate (n=3) as fixed variables, and individual mosquitoes (mosquitoID) as a random variable. The package *DHARMa* was used to check the fit of each model and to check for overdispersion (27). The package *Effects* was used to plot final GLMM model results (28, 29) and the Akaike information criterion (AIC) was used to select the best models.

For analysis of prevalence, the GLMM was fit by maximum likelihood with a binomial error distribution and logit link function using R package *lme4* (30). Infection intensity (number of oocysts per mosquito) was modelled using *glmmTMB* (31) in R with the best fit and most parsimonious model from Poisson, negative binomial, zero-inflated negative binomial and a hurdle model of the zero-inflated negative binomial defined by comparison of AIC.

## Results

### Effect of bacterial bio-products on infection prevalence

We first determined if LB medium influences the parasite infection of mosquitoes and compared the results from the two experimental controls (Supplementary Tables 2, 3). There was no difference in prevalence of infection between controls with and without re-suspended LB medium (*P*=0.18). Therefore, we proceeded to compare bacterial bio-product test groups with LB controls alone (Table 1).

**Figure 1:**
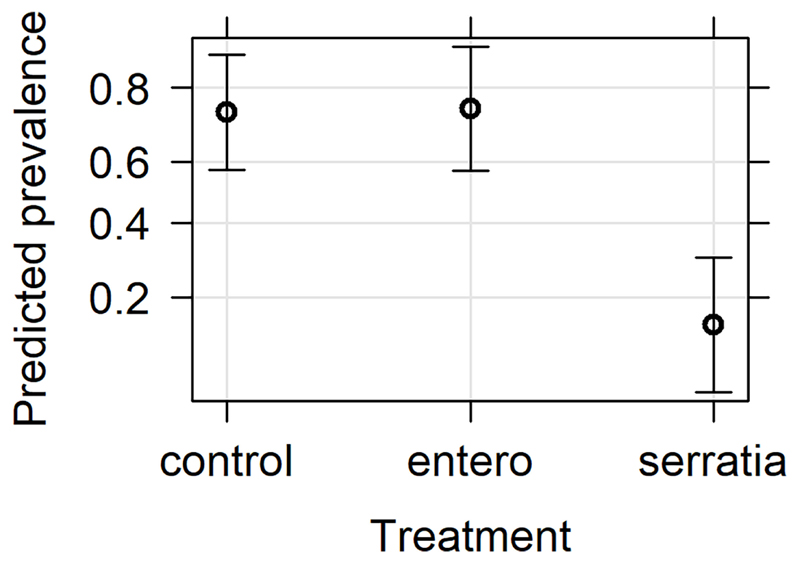
Effects plot of the generalised linear mixed model for infection prevalence following addition of bacterial bio-product to infected blood meal. The model included the fixed effects of treatment, mosquito wing length, gametocyte density in the blood meal and replicate, with the random effect of mosquito. The predicted infection prevalence from the fitted model is shown with 95% confidence intervals predicted from the model.

The effect of bio-products from *Serratia* and *Enterobacter* cultures on mosquito infection prevalence was determined using GLMM with mosquito size, gametocyte density, replicate and bio-product treatment as fixed effects. The best fit model included all fixed effects (Table 2). Bio-product from *S. marcescens* significantly reduced infection prevalence (*P*=4.02 x 10^-5^, estimate -2.818), whereas that from *E. cloacae* was similar to the LB control (*P*=0.929) (Figure 1). Infection prevalence correlated positively with mosquito size blood (winglength *P*=0.0487, estimate = 2.306), which was expected as larger mosquitoes take in larger volumes of blood and thus more gametocytes (Supplementary Figure S1A). Similarly, there was a significant positive correlation between gametocyte density in the blood meal and infection prevalence (*P*=0.0079, estimate = 0.576) (Supplementary Figure S1A).

### Effect of bacterial bio-products on oocyst intensity

**Figure 2:**
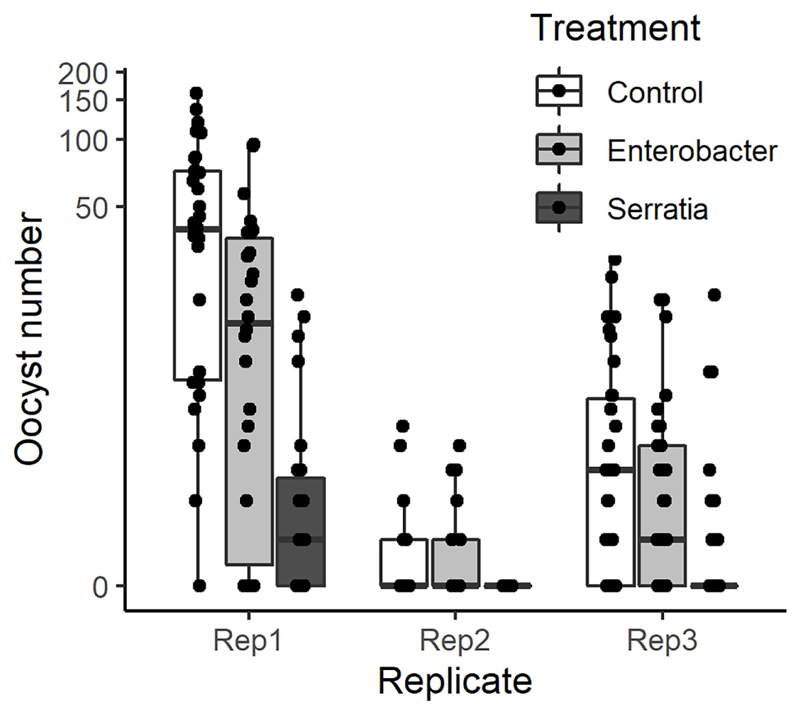
Intensity of infection (oocyst numbers) in mosquitoes fed with blood meal containing lyophilized medium from *Enterobacter* (*Ec*), *Serratia* (*Sm*) and Control (LB). Individual mosquito data points are shown as black dots. Boxes represent the 25^th^ and 75^th^ percentiles, with the median as a solid line. The whiskers represent the largest and smallest value no further than 1.5 x the interquartile range.

**Figure 3:**
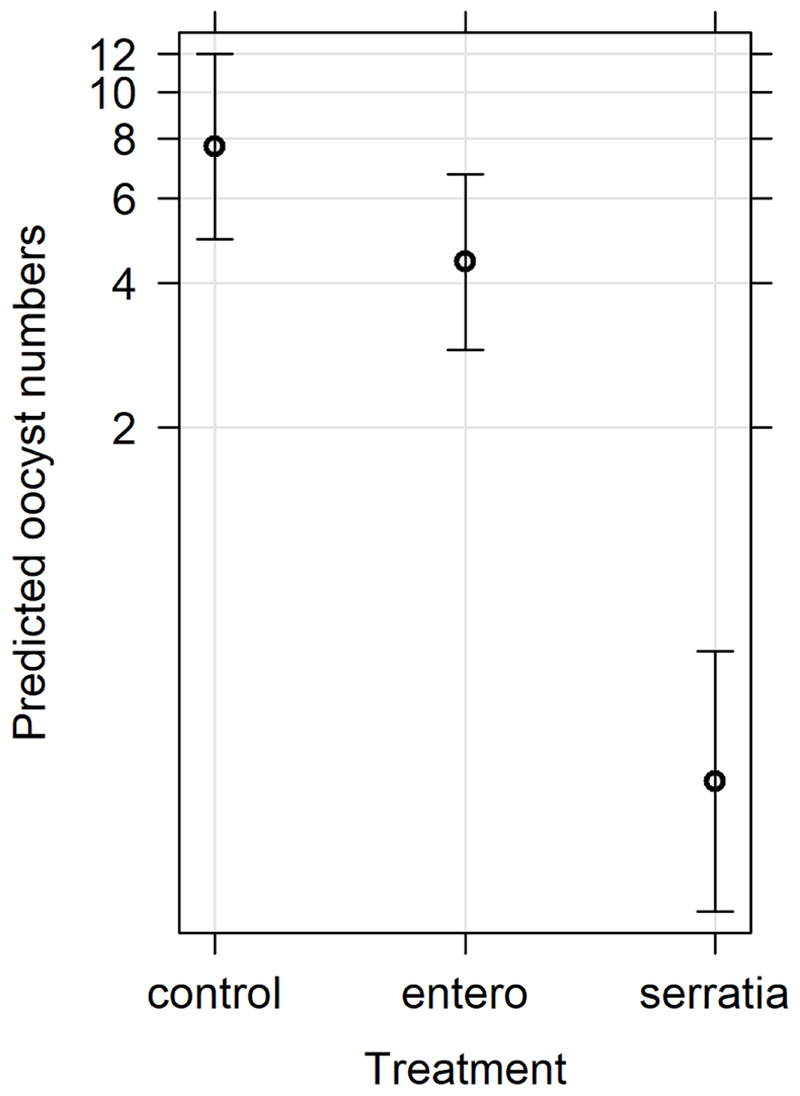
Effects plot of the generalised linear mixed model for oocyst numbers, with fixed effects of treatment and replicate, and the random effect of mosquito. The predicted oocyst numbers are shown with 95% confidence intervals predicted from the model.

The effect of *E. cloacae* and *S. marcescens* bio-product on oocyst numbers was determined by dissecting mosquitoes 10-11 days after ingesting infectious blood meal and counting oocysts (Table 1; Figure 2). Infection intensity (number of oocysts per mosquito) was modelled using a generalised linear mixed model in R, using package *glmmTMB* (31). The best fit model was a zero-inflated negative binomial model (nbinom2: variance is modelled as *μ*(1 + *μ/ϕ*)), and included the fixed effects of bio-product treatment and replicate, and mosquitoID as a random effect (Table 3). The mosquito wing length (P=0.34) and gametocyte density in the blood meal (P=0.53) were not significant predictors of oocyst intensity. As previously observed for infection prevalence, bio-product from *S. marcescens* significantly reduced oocyst intensity (*P*<2 x 10^-16^, estimate -3.0428) whereas that from *E. cloacae* did not (*P*=0.053) (Figure 3).

## Discussion

This study investigated the impact of aseptic bacterial products released into *in vitro* culture media on the mosquito stages of *P. falciparum.* Bacteria symbionts associated with mosquitoes are being intensively researched for their anti-parasitic and/or vector population suppression properties for new control methods. The two species of bacteria *Enterobacter cloacae* and *Serratia marcescens* were isolated from field-collected *An. gambiae* mosquitoes from Ghana. *Serratia* and *Enterobacter* are common bacterial species found in *Anopheles* mosquitoes, proliferate especially after blood meals (32, 33) and their impact on *Plasmodium* has been reported (11, 12, 20). These previous studies used live bacteria which were either reintroduced into mosquitoes prior to infectious blood meals or mixed with the blood meal containing the parasites (7, 11). Here we investigated if these bacteria release anti-parasitic products which can influence parasite development in mosquitoes in the absence of the bacterial cells. The material released into culture medium by the *S. marcescens* isolate was shown to reduce significantly both the prevalence and infection intensity of *P. falciparum* in *An. gambiae* mosquitoes. The blocking effect with *S. marcescens* product was observed in all experimental replicates with different infection prevalence and intensity. At lower prevalence in replicate 2, where infection prevalence in the control was 36%, *S. marcescens* product completely blocked mosquito infection. Despite the difference in the parasite infection levels obtained in the replicates, all three showed similar bacterial-mediated oocyst reduction effects. Variation in parasite intensity is a natural phenomenon and within the limits of this study, we have shown that the microbial effect may not be dependent on the parasite infection intensity. Although this is promising for control, additional studies will, however, be required to confirm these results.

All transmission reduction activity observed in this study occurred in mosquitoes with intact natural microbiota, suggesting a direct effect on the parasites, rather than through enhanced triggering of mosquito immune responses. It is also possible that the lyophilate could augment the mosquito immune response to the naturally occurring bacteria, or could affect their growth, resulting in lower infection levels. Many studies have focused on the innate immune system because as mosquitoes take in a blood meal, there is an increase in the microbial community in the midgut (12, 34, 35), which activate signalling pathways such as the Immune Deficiency (IMD) and Toll pathways, and reduce *Plasmodium* numbers in the midgut (10, 36). Experiments to assess bacterial effects on mosquito physiological processes and *Plasmodium* development often use aseptic mosquitoes that were cleared of residing bacteria by introduction of antibiotics through sugar-feeding (7, 12, 37, 38) or through creating axenic mosquitoes (39, 40). Using this aseptic procedure, an isolate of *S. marcescens* from a laboratory colony of *An. gambiae* effected reduction in *P. falciparum* infection intensity (12). A similar impact on *P. berghei* infection intensity was observed with *S. marcescens* adapted from field-caught mosquitoes in Burkina Faso (20). However the absence of competing natural bacteria in this experimental system may result in higher colony forming units of re-introduced bacteria. *In vitro* development of *P. berghei* ookinetes was reduced in the presence of *S. marcescens* (12, 20) but also with supernatants from bacterial liquid cultures (12), suggesting that secreted factors were involved in direct killing of ookinetes and oocysts. A related species, *S. ureilytica,* has been shown to produce an antimalarial lipase which can kill the parasite at the gametocyte and ookinete stages. Immunofluorescence assays revealed the lipase was detected on *P. berghei* ookinetes, suggesting a direct killing mechanism (15). Our study confirms the anti-Plasmodial activity of secreted products from *S. marcescens* and further shows their effectiveness without clearing or reducing the host microbial community. Further studies will however be required to demonstrate whether the anti-parasite mechanism of *S. marcescens* is solely due to the direct action of bacterial secreted products on the parasites (7, 41), or if recognition of *S. marcescens* in the midgut by the mosquito immune system and subsequent enhancement of antibacterial effectors also contributes to killing parasites.

We did not observe any significant transmission blocking activity with bioproducts from *Enterobacter cloacae.* Previous studies demonstrated that *Enterobacter (Esp_Z)* isolated from *An. arabiensis* in Zambia negatively affected ookinete development, but the anti-parasitic effect was reduced when the bacteria were heat-inactivated before re-introduction into mosquitoes (11). Filtered fresh *Esp_Z* culture supernatant was able to reduce *P. berghei* ookinete formation *in vitro*, and the authors concluded that parasite inhibition was mediated by production of reactive oxygen species (ROS), a short-lived bacterial-produced molecule. The lack of anti-parasite activity with *E. cloacae* culture supernatant in the current study could be because ROS may have been depleted at our 8-hour harvesting of bacterial bio-product or destroyed during the lyophilization process. Therefore, further investigations will be needed to determine if collection of supernatant at different time points (growth stages) of culture will impact on *Plasmodium* development, and/or if this *E. cloacae* isolate from *An. gambiae s.l* mosquitoes collected from Ghana produces ROS, as reported for *Esp_Z* (11).

## Supplementary Material

The Supplementary Material for this article can be found online at: https://www.frontiersin.org/articles/10.3389/fitd.2022.979615/full#supplementary-material

Supplementary Information

## Figures and Tables

**Table 1:** Summary of *P. falciparum* infection in experimental groups.

	Replicate 1			Replicate 2			Replicate 3		
	LB	*Ec*	*Sm*	LB	*Ec*	*Sm*	LB	*Ec*	*Sm*
**Number of infected mosquitoes/total dissected**	29/30	22/30	18/30	9/25	10/25	0/22	27/38	19/30	11/51
**% Prevalence**	96.7	73.3	60	36	40	0	71.1	63.3	21.6
**Range of oocyst numbers**	0-161	0-95	0-10	0-5	0-4	0	0-73	0-19	0-20
**Median of oocyst numbers**	39.5	15	1	0	0	0	3	1	0
**Mean mosquito wing length in mm (sem)**	2.41	2.57	2.53	1.64	1.74	1.66	2.78	2.78	2.84
	(0.029)	(0.033)	(0.031)	(0.030)	(0.027)	(0.026)	(0.026)	(0.024)	(0.022)

Prevalence, oocyst intensity and body size measurements are shown for mosquitoes treated with re-suspended LB, and bio-products from *S. marcescens* (*Sm*) and *E. cloacae* (*Ec*). Numbers in brackets are standard error of mean (sem).

**Table 2:** Summary of significant variables from a generalised linear mixed model (GLMM) predicting parasite prevalence.

	Estimate	Std. error	z value	*P*-value^1^
Intercept	-5.99378	2.25198	-2.662	0.00778**
Treatment (*Ec* vs LB)	0.04413	0.49734	0.089	0.92929
Treatment (*Sm* vs LB)	-2.81805	0.68624	-4.107	4.02e-05***
Wing length	2.30606	1.16965	1.972	0.04866*
Gametocyte density	0.57583	0.21681	2.656	0.00791**
Replicate (Rep 2 vs Rep 1)	1.15147	1.29483	-0.88	0.37385
Replicate (Rep 3 vs Rep 1)	4.05823	1.13457	3.577	0.000348***
Replicate (Rep 2 vs Rep 3)	-5.20972	2.19677	-2.372	0.01771*

1 Signif. codes:

*** <0.001;

** <0.01;

* < 0.05. LB, control LB treatment; Ec, *E. cloacae* product treatment; Sm, *S. marcescens* product treatment.

**Table 3:** Summary of significant variables from a zero-inflated negative binomial generalised linear mixed model (GLMM) predicting oocyst intensity.

	Estimate	Std. error	z value	P-value^1^
Intercept	-1.4231	1.3034	-1.092	0.2749
Treatment (Entero vs control)	-0.5521	0.2852	-1.935	0.0529
Treatment (Serratia vs control)	-3.0428	0.3322	-9.161	< 2e-16***
Replicate	-3.9480	0.3015	-13.095	< 2e-16***
Replicate (Rep 2 vs Rep 1)	3.4478	0.6888	5.006	5.57e-07***
Replicate (Rep 2 vs Rep 3)	1.3318	0.8773	1.518	0.1290
Replicate (Rep 1 vs Rep 3)	-2.1160	0.3439	-6.152	7.63e-10***

1 Signif. codes:

*** < 0.001

** < 0.01

* <0.05. LB, control LB treatment; Ec, *E. cloacae* product treatment; Sm, *S. marcescens* product treatment.

## Data Availability

The original contributions presented in the study are included in the article/Supplementary Material. Further inquiries can be directed to the corresponding authors.
