# Supplementary material for: Bio-products from Serratia marcescens isolated from Ghanaian Anopheles gambiae reduce Plasmodium falciparum burden in vector mosquitoes
Source: Front Trop Dis. Author manuscript; Available in PMC 2023 Feb 3. (PMC7614139; doi:10.3389/fitd.2022.979615)
Supplement: Supplementary Information [file EMS162590-supplement-Supplementary_Information.pdf]

# Supplementary Information

### Bio-products from *Serratia marcescens* isolated from Ghanaian *Anopheles gambiae* reduce *Plasmodium falciparum* burden in vector mosquitoes

Esinam Abla Akorli[1,2], Prince Chigozirim Ubiaru[2], Sabyasachi Pradhan[2], Jewelna Akorli[1*], Lisa Ranford-Cartwright[2*]

## Supplementary Tables

**Table 1: Confirmation of cell-free spent medium.** Optical density (OD) was measured at before and after filtration to show absence of cells.

| Spent Medium | Absorbance ($OD_{600}$) | |
|---|---|---|
| | **Before filtration** | **After filtration** |
| LB | 0.001 | 0.000 |
| *Sm* | 0.567 | 0.001 |
| *Ec* | 0.529 | 0.001 |

**Table 2: Summary infection data of Control vs LB.** Number in brackets are standard error of mean.

| | **Control** | **LB** |
|---|---|---|
| Number of infected mosquitoes/ total dissected | 26/30 | 29/30 |
| % Prevalence | 86.7 | 96.7 |
| Range of oocyst numbers | 0-147 | 0-161 |
| Median of oocyst numbers | 49 | 39.5 |
| Mean mosquito wing length in mm (sem) | 2.45 (0.04) | 2.41 (0.029) |

**Table 3**: Summary of binomial generalised linear model (GLM) results between controls. Mosquito body size (mosquito wing length) was included in the comparison.

| | **Estimate** | **Std. Error** | **Z value** | ***P*-value** |
|---|---|---|---|---|
| Control_LB | 1.563 | 1.176 | 1.33 | 0.1836 |
| Wing length | 4.278 | 2.488 | 1.72 | 0.0855 |

**Supplementary Figures**

**A** **B**

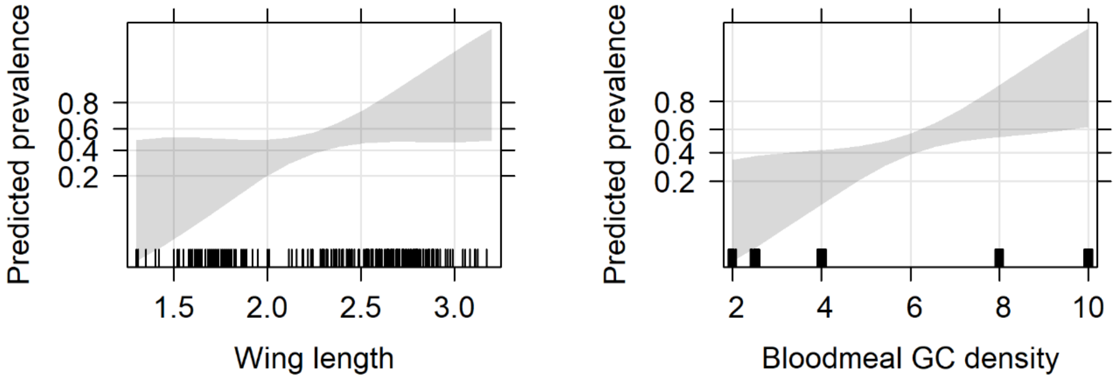


**Figure S1: Effect plots of generalised linear mixed model for infection prevalence.** The model included the fixed effects of treatment, mosquito wing length (mm), gametocyte density (gametocytes per 1000 RBC) in the blood meal and replicate, with random effect of mosquito. The predicted infection prevalence from the fitted model is shown with 95% confidence intervals predicted from the model (blue ribbons) for the fixed effects of (A) wing length; (B) gametocyte density in the blood meal.

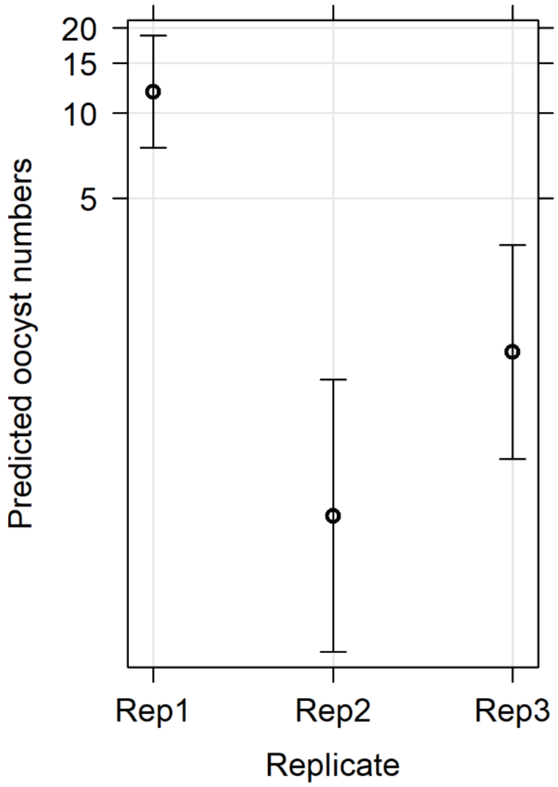


**Figure S2: Effects plot of generalised linear mixed model for oocyst numbers (infection intensity).** The model included the fixed effects of treatment, and replicate, with random effect of mosquito**.** The predicted oocyst numbers are shown with 95% confidence intervals predicted from the model for the fixed effect of replicate.
